# Influence of intrapatient variability in tacrolimus trough levels on acute rejection in pediatric kidney transplant recipients

**DOI:** 10.1007/s00467-025-07028-1

**Published:** 2025-12-22

**Authors:** Fatina I. Fadel, Samuel H. Makar, Esraa Ehab Abbas, Mahmoud Ibrahim Mostafa, Mohamed Ahmed Mobarez, Shorouk A. Othman

**Affiliations:** 1 Pediatric Nephrology and Transplantation Unit, Department of Pediatrics, Kasr Alainy Faculty of Medicine, Cairo University, Cairo, Egypt; 2 Division of Pharmacy Practice, Faculty of Pharmacy, Helwan University, Cairo, Egypt

**Keywords:** Pediatric kidney transplantation, Tacrolimus, Time in therapeutic range, Trough level, Acute rejection

## Abstract

**Background:**

Tacrolimus is a cornerstone of lifelong immunosuppressive therapy to prevent acute rejection post-kidney transplantation. Tacrolimus intra-patient variability (IPV) is characterized by several pharmacokinetic metrics, including the standard deviation (SD) of tacrolimus troughs, coefficient of variation (CV%), dose-normalized concentration (DNC), and time in therapeutic range (TTR). This study aimed to investigate the influence of TTR, alongside other IPV metrics, on the incidence of acute rejection in the first year after kidney transplantation.

**Methods:**

This single-center retrospective study evaluated the relationship between IPV measures including coefficient of variation (CV%), standard deviation (SD), dose-normalized concentration (DNC), time in therapeutic range (TTR), and acute rejection during the first post-transplant year in 100 pediatric kidney recipients.

**Results:**

Patients were stratified by TTR into two subgroups: TTR < 78% (*n* = 80) and TTR ≥ 78% (*n* = 20). The mean CV% of tacrolimus concentration was 37.1 ± 16.6%, with significantly higher variability observed in those with rejection (*p* = 0.031). Longitudinal analysis showed that differences in trough levels between TTR groups became evident after 3 months (*p* < 0.001). Multivariable modeling demonstrated that rejection risk was independently associated with higher age (*p* = 0.002) and post-transplant period beyond 3 months (*p* = 0.004), rather than TTR itself.

**Conclusions:**

In pediatric kidney transplant patients, the rejection risk was significantly associated with the magnitude of CV% rather than TTR. Special attention is warranted for therapeutic drug monitoring, especially beyond 3 months post-transplant, due to the increased risk of rejection compared to earlier stages post-transplantation.

**Graphical abstract:**

A higher resolution version of the Graphical abstract is available as Supplementary information

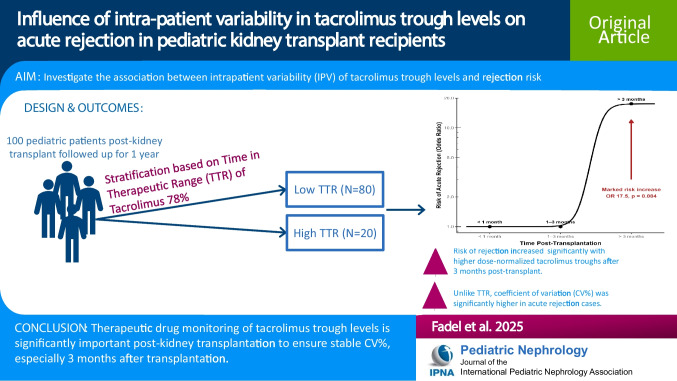

**Supplementary information:**

The online version contains supplementary material available at 10.1007/s00467-025-07028-1.

## Introduction

Kidney transplantation is considered the optimal treatment option for individuals with kidney failure [1]. Graft survival has improved dramatically over the past three decades. It is now evident that long-term graft survival is steadily improving [2]. The primary goal of immunosuppressive therapy is to prevent acute rejection while minimizing side effects [3]. The most commonly used maintenance immunosuppressive regimen consists of a calcineurin inhibitor (CNI) most often tacrolimus combined with mycophenolate mofetil (MMF) and glucocorticoids [4, 5].

Tacrolimus is a potent CNI that is widely regarded as the keystone of maintenance immunosuppressive therapy to prevent acute allograft rejection after kidney transplantation. Its clinical benefits in improving kidney transplantation outcomes are well documented [6]. However, due to its narrow therapeutic window and significant pharmacokinetic variability, frequent and individualized dose adjustments are crucial to prevent potential treatment failures, including graft rejection and adverse effects [7].

Tacrolimus intrapatient variability (IPV) reflects fluctuations in tacrolimus trough level within a patient over a specific time period [8, 9]. IPV can be quantified using various metrics such as standard deviation (SD) [10, 12], coefficient of variation (CV%) [11, 13, 14], the concentration/dose ratio (C/D ratio) or dose-normalized concentrations (DNC) [15], and time in therapeutic range (TTR) [16]. TTR is defined as the percentage of time a patient’s trough levels remain within target range, taking stability, intensity, and constancy into consideration simultaneously [17]. Each of these measures yields distinct insights. Currently, there is no consensus on which of these metrics is considered the most appropriate in characterizing tacrolimus IPV or its impact on kidney transplantation outcomes.

Many reports have demonstrated that IPV in tacrolimus trough levels, often expressed as CV%, are associated with increased risk of donor-specific antibody development and several other adverse outcomes in adult transplant recipients [15, 18, 19]. Other studies suggested that TTR represents a useful tool to be considered alongside individual tacrolimus trough levels [16]. However, most available evidence was derived from adult populations, with limited data in pediatric patients.

Therefore, this study aimed to identify the potential associations between IPV/exposure metrics and the incidence of acute rejection in pediatric patients during the first year after kidney transplantation. To the best of our knowledge, this is the first study to address this issue in the context of pediatric kidney transplantation.

## Patients and methods

### Study design

We conducted a single-center, retrospective cohort study involving pediatric patients who underwent kidney transplantation at the kidney transplantation unit of Cairo University Children’s Hospital between June 2019 and October 2024 and subsequently received tacrolimus as part of their immunosuppressive regimen in the first year following transplantation.

### Patients

A total of 100 patients of both sexes, aged up to 15 years, were included. Patients who received multi-organ transplants, underwent retransplantation, received nonstandard immunosuppressive regimens, and deviated from standard tacrolimus therapy were nonadherent to treatment or were concomitantly receiving medications known to affect tacrolimus metabolism (e.g., fluconazole, clarithromycin, diltiazem, or verapamil) were excluded.

Nonadherence was assessed using two objective measures: (1) medication possession ratio (MPR) calculated from pharmacy refill records, with < 80% defined as nonadherent, and (2) clinic attendance records, with ≥ 2 consecutive missed visits defined as nonadherent. Patients who satisfied either of the two criteria were classified as nonadherent.

### Data collection

Data were collected from patient records at the kidney transplantation clinic. For each patient, we obtained demographic characteristics, clinical history (including the underlying kidney disease, duration of hemodialysis before transplantation, and donor characteristics), cytomegalovirus (CMV) serostatus, induction therapy, immunosuppressive regimen, and details of acute rejection episodes (including date and type of rejection). Laboratory investigations, including complete blood count, kidney and liver function tests, urine albumin-to-creatinine ratio, urinalysis, CMV polymerase chain reaction, and tacrolimus trough levels, were collected at regular intervals during the first year post-transplantation.

### Tacrolimus monitoring and pharmacokinetic parameters

Tacrolimus trough levels were measured in blood samples collected at regular intervals during the first post-transplantation year. Monitoring frequency followed a standardized protocol, with trough concentrations obtained weekly during the first month, biweekly during months 2 and 3, and monthly thereafter. Additional levels were drawn for clinical indication, including suspected rejection or toxicity. All samples were collected 30 ± 5 min before the morning dose and processed within 2 h of collection. Analysis of all samples was performed in the central laboratory of Cairo University Children’s Hospital using the enzyme multiplied immunoassay technique (EMIT). All assays were conducted by the same personnel to minimize inter-observer variability. During the study period, goal tacrolimus trough level was consistently set at 8–12 ng/ml during month 1, 6–9 ng/ml during months 2–3, and 4–8 ng/ml from month 4 onwards, in accordance with the Egyptian Society of Pediatric Nephrology and Transplantation (ESPNT) 2019 local protocol.

Several pharmacokinetic parameters were calculated to characterize tacrolimus exposure and IPV, including the percent TTR, CV% of tacrolimus concentrations, DNC, mean tacrolimus concentration, mean DNC, and SD of tacrolimus concentrations. The TTR was calculated using the Rosendaal method for linear interpolation [20]. This method assumes a linear relationship between two consecutive tacrolimus measurements and estimates the time spent within the therapeutic range. Patients were categorized into two groups based on a TTR cutoff of 78%, as previously suggested by Song et al. [17]. TTR was calculated for each patient over the entire study period and separately for three time intervals: < 1 month, 1–3 months, and > 3 months after transplantation. The SD of tacrolimus concentrations was calculated to quantify the variability in tacrolimus trough concentrations within each patient. The CV% was computed as (SD/mean) × 100 to provide a normalized measure of tacrolimus level variability per patient. DNC was calculated by dividing the measured tacrolimus trough concentration by the corresponding daily dose. The mean tacrolimus concentration was estimated as the arithmetic mean of all available tacrolimus trough concentrations for each patient over the study period. Similarly, the mean DNC was determined as the arithmetic mean of all calculated DNC for each patient.

### Outcome measures

The primary outcome of this study was the occurrence of acute rejection, defined as an acute decline in graft function, typically manifested by an increase in serum creatinine and confirmed by histopathological evidence of immune-mediated graft injury on biopsy. Acute rejection included both clinically apparent T-cell-mediated rejection (TCMR) and antibody-mediated rejection (ABMR), diagnosed according to the Banff criteria [21]. Alternative causes of graft dysfunction, including infection, sepsis, and dehydration, were excluded. The secondary outcome was the association of the investigated pharmacokinetic parameters of intra-patient variability and the risk of acute rejection.

### Sample size

We calculated the sample size based on the assumption of a 27% difference in acute rejection rate between patients with low versus high tacrolimus TTR, based on a previous study [17]. Using a two-sided alpha of 0.05 and 80% power, a minimum of 42 patients was required in each group (84 in total), based on the two-sample test of proportions. To account for an anticipated 15% rate of missing data, we increased the sample size to a minimum of 97 patients.

### Statistical analysis

Continuous variables were summarized as means with standard deviations (SD) or medians with interquartile ranges (IQRs), based on their distribution. Categorical variables were presented as counts and percentages (*n*, %). Normality was assessed using the Shapiro–Wilk test and visual inspection of histograms. Comparisons between groups (TTR < 78% vs. ≥ 78%, and rejection vs. no rejection) for continuous variables were conducted using the Wilcoxon rank sum test. For categorical variables, Pearson chi-squared test or Fisher’s exact test was used as appropriate, based on expected cell counts. Changes in tacrolimus parameters across time intervals (< 1 month, 1–3 months, > 3 months post-transplantation) were analyzed using the Friedman ANOVA test. To identify factors associated with rejection risk, univariate mixed-effects logistic regression models were employed. The mixed-effects approach accounted for repeated measures within patients over different time intervals. Kaplan–Meier analysis was used to estimate 1-year rejection-free survival, stratified by TTR group (< 78% vs. ≥ 78%). Survival curves were compared using the log-rank test. All statistical tests were two-sided, with a significance level of *α* = 0.05. Analyses were performed using R version 4.3.0 (R Foundation for Statistical Computing, Vienna, Austria).

## Results

### Patient selection and baseline characteristics

Of the 100 pediatric kidney transplant recipients included in the study, 80 patients (80%) had a TTR less than 78%, and 20 patients (20%) had a TTR of 78% or greater. The mean age of the patients was 10.8 ± 3.8 years, with no significant differences between TTR groups (*p* = 0.5). Male recipients predominated, comprising 70% of the study population, with a comparable distribution between TTR groups (72.5% vs. 60.0%, *p* = 0.3).

All transplants were performed using living-related donors, with mothers representing 62% of donors. The mean donor age was 37 ± 6.6 years. The median duration of pre-transplant hemodialysis for the recipients was 10 months (interquartile range [IQR], 1 to 17.3), with no significant differences between groups. Human leukocyte antigen (HLA) mismatch scores were comparable between groups (*p* = 0.6). The majority of our cases (50.80%) demonstrated 3 to 4 HLA mismatches. Focal segmental glomerulosclerosis (FSGS) was the most common primary kidney disease overall (26%), followed by bilateral atrophic kidney (20%). However, the distribution of primary diagnoses varied slightly between TTR groups, with FSGS being more prevalent in the low TTR group (28% vs. 20%) and bilateral atrophic kidney more common in the high TTR group (16% vs. 35.0%), though these differences did not reach statistical significance (*p* = 0.065) (Table 1).

**Table 1:** Baseline demographic, clinical, laboratory, and treatment characteristics stratified by tacrolimus TTR

Characteristic	Overall (*N* = 100)	TTR < 78% (*N* = 80)	TTR ≥ 78% (*N* = 20)	*p*
**Demographic and clinical characteristics**				
Age (years), mean ± SD	10.8 ± 3.8	10.8 ± 3.9	10.5 ± 3.8	0.5^a^
Female sex, *n* (%)	30 (30.0)	22 (27.5)	8 (40.0)	0.3^b^
Weight (kg), mean ± SD	28.0 ± 10.3	28.1 ± 10.4	27.6 ± 10.2	0.9^a^
Donor age (years), mean ± SD	37.0 ± 6.6	37.5 ± 6.8	35.3 ± 5.6	0.2^a^
Donor female sex, *n* (%)	62 (62.0)	49 (61.3)	13 (65.0)	0.8^b^
Mother as donor, *n* (%)	62 (62.0)	49 (61.3)	13 (65.0)	0.8^b^
Number of HLA mismatch				0.6^c^
No mismatch	2 (3.2%)	2 (3.8%)	0 (0%)	
I	6 (9.5%)	5 (9.4%)	1 (10%)	
II	23 (37%)	21 (40%)	2 (20%)	
III	29 (46%)	22 (42%)	7 (70%)	
IV	3 (4.8%)	3 (5.7%)	0 (0%)	
Hemodialysis duration (months), median (IQR)	10.0 (1.0–17.3)	10.0 (1.8–18.0)	10.0 (1.0–12.0)	0.9^a^
**Primary renal disease, *n* (%)**				
FSGS	26 (26%)	22 (28%)	4 (20%)	0.065^c^
Bilateral atrophic kidney	20 (20%)	13 (16%)	7 (35%)	
CAKUT	19 (19%)	18 (23%)	1 (5.0%)	
Cystic kidney disease	15 (15%)	10 (13%)	5 (25%)	
Neurogenic bladder	11 (11%)	8 (10%)	3 (15%)	
Others	9 (9.0%)	9 (11%)	0 (0%)	
**Laboratory parameters**				
Creatinine (mg/dL), median (IQR)	0.7 (0.5–0.9)	0.7 (0.5–0.9)	0.9 (0.6–0.9)	0.2^a^
Urea (mg/dL), median (IQR)	30.0 (24.0, 52.0)	30.0 (23.0, 49.5)	47.5 (41.3, 53.8)	0.4^a^
Hemoglobin (g/dL), mean ± SD	10.8 ± 1.7	10.8 ± 1.7	11.1 ± 1.5	0.4^a^
Total leukocyte count (× 10^3^/mm^3^), mean ± SD	9.0 (6.0, 12.0)	8.6 (6.0, 11.3)	10.0 (8.1, 13.6)	0.1^a^
Platelet count (× 10^3^/mm^3^), mean ± SD	287.5 (200.0, 401.3)	262.5 (199.5, 401.3)	346.0 (269.5, 402.0)	0.3^a^
AST (U/L), median (IQR)	25.0 (17.0–43.0)	24.0 (16.0–36.0)	34.0 (24.3–63.3)	0.3^a^
ALT (U/L), median (IQR)	23.0 (17.0–54.0)	23.0 (18.0–54.0)	17.0 (13.8–27.3)	0.4^a^
CRP (mg/L), median (IQR)	6.0 (2.0, 6.0)	6.0 (2.0, 10.5)	6.0 (0.0, 6.0)	0.5^a^
Albumin/creatinine ratio (median (IQR)	324.0 (48.0, 1,158.0)	275.0 (56.0, 1,190.0)	342.0 (111.0, 934.5)	> 0.9^a^
Urinary RBC (cells/hpf), mean ± SD	42.9 ± 84.6	52.4 ± 91.2	0.0 ± 0.0	**0.04**^**a**^
**Immunosuppressive treatment, *n* (%)**				
Induction therapy				0.7^c^
ATG + methylprednisolone	71 (71.0)	56 (70.0)	15 (75.0)	
Basiliximab + methylprednisolone	29 (29.0)	24 (30.0)	5 (25.0)	
Maintenance therapy				
Mycophenolate mofetil	37 (37.4)	28 (35.4)	9 (45.0)	0.4^b^
Mycophenolic acid	58 (58.0)	47 (58.8)	11 (55.0)	0.8^b^
Azathioprine	1 (1.0)	1 (1.3)	0 (0.0)	> 0.9^c^
Oral prednisolone	86 (86.9)	68 (86.1)	18 (90.0)	> 0.9^c^
Antihypertensive medication	16 (16.0)	11 (13.8)	5 (25.0)	0.3^c^

^a^Wilcoxon rank sum test

^b^Pearson chi-squared test

^c^Fisher’s exact test

*SD*, standard deviation; *IQR*, interquartile range; *FSGS*, focal segmental glomerulosclerosis; *CAKUT*, congenital anomaly of kidney and urinary tract; *AST*, aspartate aminotransferase; *ALT*, alanine aminotransferase; *CRP*, C-reactive protein; *RBC*, red blood cells; *hpf*, high power field; *ATG*, anti-thymocyte globulin; *HLA*, human leukocyte antigen

Bold entries indicate significant findings

Laboratory values were generally similar between groups, except for the urinary red blood cell count, which was significantly higher in the low TTR group (52.4 ± 91.2 vs. 0 ± 0 cells per high-power field, *p* = 0.04). Albuminuria was more frequently detected in the low TTR group; however, the difference was not statistically significant (*p* < 0.9). Serum creatinine levels were within the normal range for both groups (Median 0.7 mg/dl; IQR, 0.5 to 0.9) (Table 1).

The immunosuppression protocol was consistent across groups. Most patients (71%) received depleting induction therapy—antithymocyte globulin (ATG) plus methylprednisolone—while the remainder (29%) received a non-depleting agent, basiliximab plus methylprednisolone. Maintenance immunosuppression primarily consisted of tacrolimus combined with either mycophenolic acid (58%) or mycophenolate mofetil (37.4%), along with oral prednisolone (86.9%). The type of maintenance agents did not differ significantly between TTR groups (Table 1).

### Rejection patterns

During the 1-year follow-up period, 46 patients (46%) experienced at least one episode of allograft rejection, with similar frequencies observed between the TTR groups (46.3% in TTR < 78% vs. 45.0% in TTR ≥ 78%, *p* = 0.9). The mean number of rejection events per patient was 1.3 ± 2.0, resulting in comparable incidence rates between groups (1.24 vs. 1.20 events per patient, *p* = 0.89). TCMR predominated, accounting for 82.9% of all rejection episodes, with a slightly, but non-significantly, higher prevalence in the high TTR group (95.8% vs. 79.8%, *p* = 0.07) (Table 2). One-year rejection-free survival was similar between groups (54% [95% CI, 44–66] for TTR < 78% vs. 55% [95% CI, 37–82] for TTR ≥ 78%, *p* = 0.8).

**Table 2:** Rejection outcomes stratified by TTR groups

Characteristic	Overall (*N* = 100)	TTR < 78% (*N* = 80)	TTR ≥ 78% (*N* = 20)	*p*
Rejection occurrence, *n* (%)	46 (46.0)	37 (46.3)	9 (45.0)	0.9^a^
Number of rejection events	1.3 ± 2.0	1.3 ± 2.0	1.2 ± 1.9	0.8^b^
Incidence rate^c^	1.23	1.24	1.20	0.89^d^
**Rejection type**				
TCMR	102 (82.9)	79 (79.8)	23 (95.8)	0.07^e^
ABMR	21 (17.1)	20 (20.2)	1 (4.2)	
1-year rejection-free survival (95% CI)^f^	54 (45–65)	54 (44–66)	55 (37–82)	0.8^a^

^a^Pearson’s chi-squared test

^b^Wilcoxon rank sum test

^c^Incidence rate: number of events per patient

^d^Poisson regression for incidence rate differences

^e^Fisher’s exact test

^f^Percent was calculated as the proportion of rejection event type out of total events (not patients) per group

*TTR*, time in therapeutic range; *SD*, standard deviation; *CI*, confidence interval; *TCMR*, T-cell-mediated rejection; *ABMR*, antibody mediated rejection

### Tacrolimus therapy characteristics

Initial tacrolimus dosing was comparable between TTR groups (mean dose, 5.8 ± 2.6 mg vs. 5.3 ± 1.8 mg; *p* = 0.5) and between patients who did and did not experience rejection (6 ± 2.7 mg vs. 5.5 ± 2.2 mg; *p* = 0.5). Patients required a mean of 4.3 ± 2.1 dose adjustments during the study period, with no significant differences based on TTR or rejection status. Similarly, the patients received a mean dose of 5.9 ± 2 mg over the complete study period, with no significant differences attributed to TTR stratum or rejection status (Table 3).

**Table 3:** Tacrolimus therapy characteristics stratified by TTR groups and rejection status

Variable	Overall (*N* = 100)	TTR status			Rejection status		
		**TTR < 78% (*N* = 80)**	**TTR ≥ 78% (*N* = 20)**	***p***^**a**^	**No rejection (*N* = 54)**	**Rejection (*N* = 46)**	***p***^**a**^
**Initial parameters**							
Initial dose (mg)	5.7 ± 2.4	5.8 ± 2.6	5.3 ± 1.8	0.5	5.5 ± 2.2	6.0 ± 2.7	0.5
Dose adjustments (*n*)	4.3 ± 2.1	4.3 ± 2.1	4.4 ± 2.1	0.8	4.0 ± 2.0	4.7 ± 2.1	0.12
Mean dose (mg)	5.9 ± 2.0	6.0 ± 2.1	5.5 ± 1.3	0.3	5.8 ± 1.9	6.0 ± 2.0	0.7
**Concentration parameters**							
Mean concentration (ng/mL)	8.1 ± 1.5	8.4 ± 1.5	7.2 ± 0.9	** < 0.001**	8.1 ± 1.5	8.2 ± 1.4	0.7
Concentration SD (ng/mL)	3.1 ± 1.6	3.2 ± 1.7	2.3 ± 1.1	**0.008**	2.8 ± 1.6	3.3 ± 1.7	0.059
CV (%)	37.1 ± 16.6	38.4 ± 17.3	31.9 ± 13.1	0.2	34.4 ± 16.1	40.2 ± 17.0	**0.031**
DNC (ng/mL/mg)	1.7 ± 0.9	1.7 ± 1.0	1.5 ± 0.6	0.5	1.7 ± 0.8	1.7 ± 1.0	> 0.9
**Therapeutic range parameters**							
TTR (months)	5.7 ± 3.1	4.8 ± 2.7	9.3 ± 2.0	** < 0.001**	5.7 ± 3.3	5.8 ± 2.9	> 0.9
TTR (%)	54.9 ± 25.9	46.7 ± 22.1	87.6 ± 7.2	** < 0.001**	55.1 ± 26.8	54.7 ± 25.0	0.8
Time below range (%)	5.7 ± 7.9	6.1 ± 8.5	3.92 ± 4.86	0.5	4.5 ± 7.7	6.6 ± 8.0	**0.025**
Time above range (%)	39.4 ± 27.5	47.2 ± 25.1	8.47 ± 7.83	** < 0.001**	40.4 ± 29.3	38.6 ± 26.1	> 0.9

^a^Wilcoxon rank sum test

*CV*, coefficient of variation; *DNC*, dose-normalized concentration; *SD*, standard deviation; *TTR*, time in therapeutic range

Bold entries indicate significant findings

The mean tacrolimus concentration was significantly lower in the high TTR group (7.2 ± 0.9 ng/ml vs. 8.4 ± 1.5 ng/ml, *p* < 0.001), with less concentration variability (SD, 2.3 ± 1.1 vs. 3.2 ± 1.7 ng/ml, *p* = 0.008). Patients who experienced rejection showed higher CV% in tacrolimus concentrations (40.2 ± 17.0% vs. 34.4 ± 16.1%, *p* = 0.031). As expected, the high TTR group maintained therapeutic levels for a longer duration (9.3 ± 2.0 months vs. 4.8 ± 2.7 months, *p* < 0.001) and achieved a higher percentage of time within the therapeutic range (87.6 ± 7.2% vs. 46.7 ± 22.1%, *p* < 0.001). We also found a significantly larger time under therapeutic range in patients who experienced rejection compared to those who did not (6.6 ± 8.0% vs. 4.5 ± 7.7%, *p* = 0.025) (Table 3).

Comparing tacrolimus pharmacokinetic parameters across three time intervals (< 1 month, 1–3 months, and > 3 months post-transplantation) suggested distinct patterns. For TTR, significant differences in trough levels emerged after 3 months (*p* < 0.001), while dosing requirements and dose-normalized concentrations remained stable over time (Table 4). Weight-normalized tacrolimus dose varied across time points (*p* < 0.001). Compared with the first month, the mean dose increased by 0.03 mg/kg (95% CI, 0.02 to 0.05) during months 1–3 and by 0.01 mg/kg (95% CI, − 0.01 to 0.02) beyond 3 months, suggesting a consistent pattern of dose stabilization over time (Table 4). Comparing the rejection vs. non-rejection group, we found no significant differences in pharmacokinetic parameters across time intervals, and both groups exhibited similar temporal patterns.

**Table 4:** Tacrolimus therapy characteristics across different time intervals post-transplantation

Variable	< 1 month (*N* = 70)	1–3 months (*N* = 95)	> 3 months (*N* = 98)	*p*
**Drug levels and dosing**				
Tacrolimus level (ng/mL)	7.9 ± 4.5	9.0 ± 3.0	7.5 ± 1.6	**0.005**^**a**^
Tacrolimus dose (mg)	5.7 ± 2.1	6.4 ± 2.4	5.6 ± 2.1	** < 0.001**^**a**^
Tacrolimus dose (mg/kg)	0.22 ± 0.11	0.25 ± 0.12	0.23 ± 0.13	** < 0.001**^**a**^
DNC (ng/mL/mg)	1.7 ± 1.6	1.7 ± 1.5	1.6 ± 0.8	0.9^**a**^
**Variability parameters**				
Concentration SD (ng/mL)	2.7 ± 3.0	2.6 ± 2.0	2.2 ± 1.5	0.12^**a**^
CV (%)	33.0 ± 27.3	29.6 ± 17.6	27.7 ± 15.2	0.1^**a**^
**Therapeutic range parameters**				
TTR (months)	0.2 ± 0.3	0.9 ± 0.6	4.8 ± 2.9	** < 0.001**^**b,c**^
TTR (%)	36.1 ± 36.0	46.7 ± 31.2	58.4 ± 31.7	** < 0.001**^**b,c**^

^a^Mixed effects ANOVA adjusted for the number of tacrolimus troughs sampled per interval

^b^Friedman ANOVA test

^c^TTR-based measures were not adjusted for the number of samples since they were calculated from interpolated rather than actual tacrolimus concentrations

*CV*, coefficient of variation; *DNC*, dose-normalized concentration; *SD*, standard deviation; *TTR*, time in therapeutic range

Bold entries indicate significant findings

**Fig. 1:**
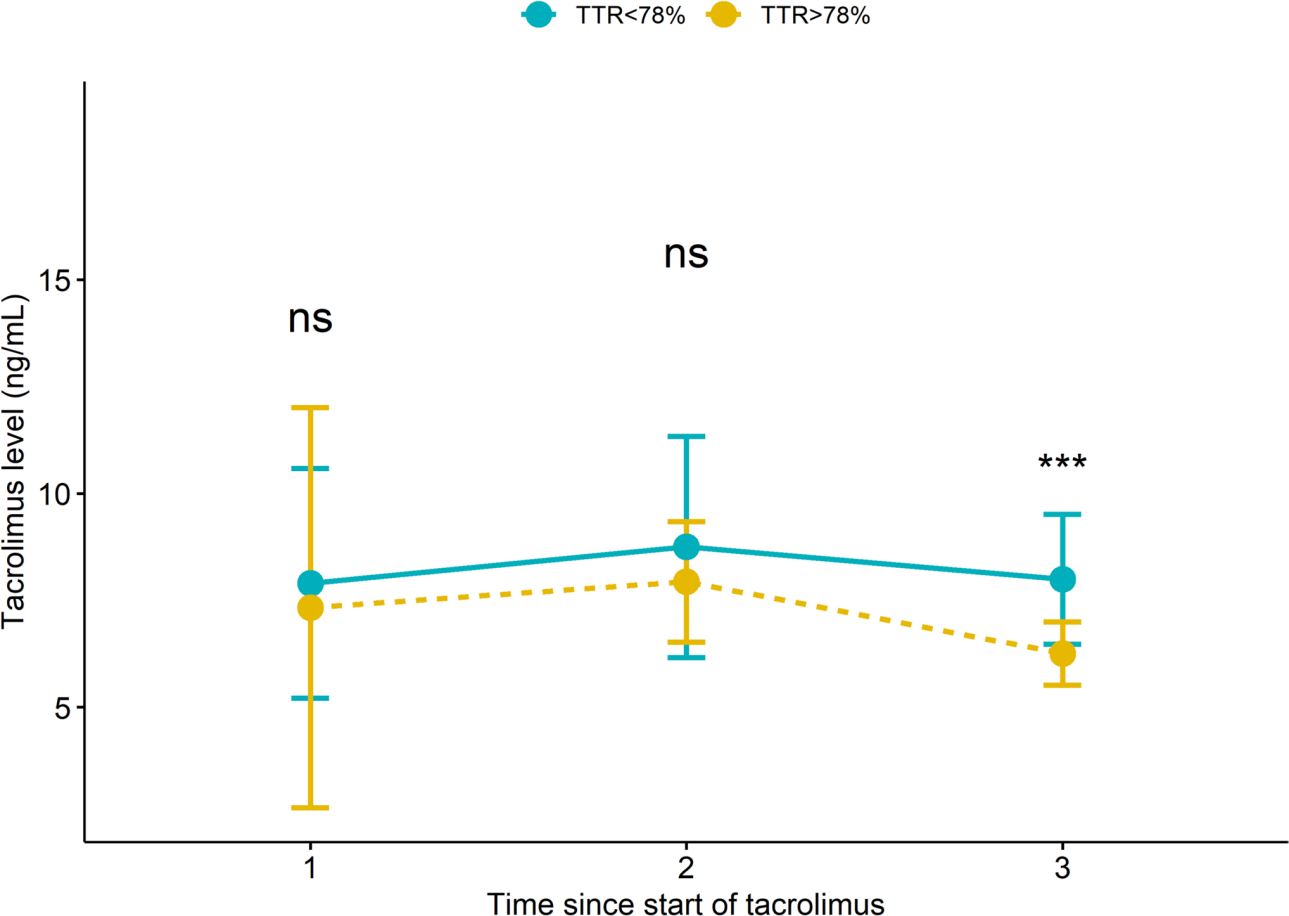
Changes in tacrolimus trough level over three time intervals (< 1 month, 1–3 months, and > 3 months) post-transplantation. It shows a comparison between TTR groups (TTR < 78% vs. TTR ≥ 78%): Data points represent the mean with error bars showing standard error. Statistical significance between groups at each time point is indicated as: ns, not significant; ***p < 0.001

TTR showed progressive improvement over the study period. The percentage of time within range increased from 36.1 ± 36.0% in the first month to 46.7 ± 31.2% at 1–3 months, reaching 58.4 ± 31.7% after 3 months (*p* < 0.001) (Table 4). By definition, the high TTR group maintained therapeutic levels for a longer duration (9.3 ± 2.0 months vs. 4.8 ± 2.7 months, *p* < 0.001) and achieved a higher percentage of time within the therapeutic range (87.6 ± 7.2% vs. 46.7 ± 22.1%, *p* < 0.001) (Table 3). Longitudinal analysis demonstrated that significant differences in trough levels between TTR groups emerged primarily after 3 months post-transplantation (*p* < 0.001) (Fig. 1), while dosing requirements and DNC remained comparable throughout the study period. Rejection status was not associated with significant differences in tacrolimus parameters at any time interval.

### Regression

We performed generalized linear mixed effects modeling to identify predictors of rejection risk within each patient during follow-up. In univariate analysis, significant predictors were patient weight (OR, 1.06; *p* = 0.026), donor age (OR, 1.13; *p* = 0.002), HLA mismatch (OR, 2.07; *p* = 0.046), and total leukocyte count (OR, 0.90; *p* = 0.014). Higher TTR was not significantly associated with reduced rejection risk (OR 0.63; *p* = 0.49 for TTR ≥ 78% vs. TTR < 78%). We found a significant interaction between DNC and post-transplant time. The impact of DNC was significant during months 1–3 post-transplantation (OR, 2.59; *p* = 0.007), but not beyond 3 months (OR, 1.00; *p* > 0.9). Rejection risk was significantly higher beyond 3 months post-transplant compared to the first month (OR, 6.59; *p* = 0.005). Other clinical characteristics, including recipient age, sex, primary kidney disease, and most laboratory parameters, showed no significant associations with rejection risk. Neither induction therapy type nor the maintenance immunosuppressive regimen influenced rejection risk (Table 5).

**Table 5:** Univariate mixed-effects logistic regression analysis of factors associated with rejection risk in pediatric renal transplant recipients

Variable	Odds ratio	95% CI	*p*-value
**Demographic and clinical characteristics**			
Age (years)	1.13	0.99, 1.29	0.072
Female sex	0.47	0.15, 1.49	0.20
Weight (kg)	1.06	1.01, 1.11	**0.026**
Donor age (years)	1.13	1.05, 1.22	**0.002**
Donor female sex	1.71	0.58, 5.00	0.33
Mother as donor	1.71	0.58, 5.00	0.33
HLA mismatch	2.07	1.01, 4.21	**0.046**
Hemodialysis duration (weeks)	0.99	0.97, 1.01	0.38
**Primary renal disease** (vs. bilateral atrophic kidney disease of unknown cause)			
CAKUT	1.00	0.21, 4.83	> 0.99
Cystic kidney disease	0.44	0.08, 2.55	0.36
FSGS	0.51	0.11, 2.32	0.38
Neurogenic bladder	2.35	0.38, 14.4	0.36
Others	0.40	0.05, 3.21	0.39
**Laboratory parameters**			
Hemoglobin (g/dL)	0.96	0.91, 1.01	0.13
Platelet count (× 10^3^/mm^3^)	1.00	1.00, 1.00	0.55
Total leukocyte count (× 10^3^/mm^3^)	0.90	0.82, 0.98	**0.014**
AST (U/L)	1.00	1.0, 1.01	0.73
ALT (U/L)	0.99	0.97, 1.00	0.11
C-reactive protein (mg/L)	0.97	0.84, 1.13	0.73
**Immunosuppressive treatment**			
Induction therapy (vs. ATG + MP)			
Basiliximab + MP	1.41	0.47, 4.29	0.54
Mycophenolate mofetil use	1.11	0.45, 2.73	0.83
Mycophenolic acid use	0.63	0.25, 1.62	0.34
Oral prednisolone use	1.15	0.29, 4.57	0.85
**Tacrolimus parameters**			
Dose (mg)	1.05	0.92, 1.20	0.48
Frequency of dose changes	1.03	0.89, 1.19	0.70
Tacrolimus concentration (ng/mL)	1.03	0.97, 1.10	0.33
Standard deviation of concentrations	1.20	0.88, 1.65	0.25
TTR ≥ 78% (vs. < 78%)	0.63	0.17, 2.33	0.49
Time interval (vs. < 1 month)			
1–3 months	0.44	0.11, 1.72	0.2
> 3 months	6.59	1.76, 24.6	**0.005**
DNC (ng/ml/mg)	0.70	0.41, 1.21	0.2
**Interaction terms**			
Dose-normalized concentration × time interval			
DNC × 1–3 months	2.59	1.30, 5.14	**0.007**
DNC × > 3 months	1.00	0.50, 2.00	> 0.9

*CI*, confidence interval; *ARPKD*, autosomal recessive polycystic kidney disease; *FSGS*, focal segmental glomerulosclerosis; *PUV*, posterior urethral valve; *AST*, aspartate aminotransferase; *ALT*, alanine aminotransferase; *ATG*, anti-thymocyte globulin; *MP*, methylprednisolone; *TTR*, time in therapeutic range; *DNC*, dose-normalized concentration

Bold entries indicate significant findings

**Fig. 2:**
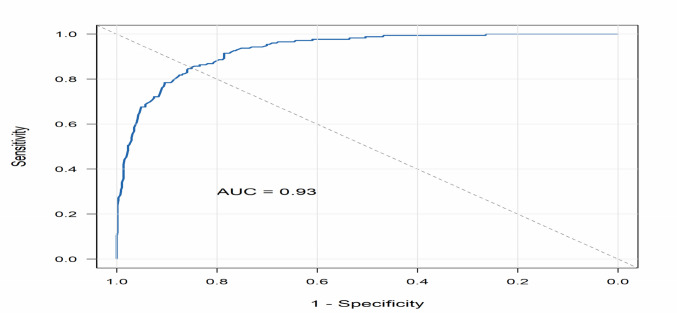
Receiver operating characteristic (ROC) curve for the generalized linear mixed model predicting the risk of rejection. The blue line represents the modelʼs discriminative ability, with an area under the curve (AUC) of 0.93. The diagonal gray dashed line indicates random chance (AUC = 0.5)

Among the significant predictors from univariate analysis, only donor age (OR, 1.14; *p* = 0.002) and post-transplant time > 3 months (OR, 17.5; *p* = 0.004, vs. first month) remained significant in multivariate analysis. The final model showed excellent discrimination for rejection risk (AUCROC = 0.93; *p* < 0.001) (Fig. 2). The final model explained 63.9% of the variance in rejection risk (pseudo *R*^2^ = 0.639) (Fig. 2).

### Sensitivity analysis

We performed sensitivity analyses to assess the robustness of our primary findings. Changing the TTR threshold from our original threshold (78%) to 60% [22] or 35% [23] did not alter the nonsignificant association between TTR and acute rejection risk. Furthermore, restricting the analysis to the stable phase (months 6–12) confirmed no significant association between IPV metrics including CV% (*p* = 0.30), SD (*p* = 0.20), or TTR (*p* = 0.60) with rejection risk. Regarding possible association between induction protocol and different IPV measures, there was no significant impact on CV% (*p* > 0.9), tacrolimus concentration ng/mL (*p* = 0.6), DNC (*p* = 0.5), and TTR (*p* = 0.8), suggesting no association between the induction protocol whether depleting or not and IPV/exposure metrics (Table 6).

**Table 6:** Sensitivity analysis

Analysis	Comparison/variable	Effect size (95% CI)	*p* value	Conclusion
**Changing TTR thresholds and risk of acute rejection**^a^	TTR ≥ 35% vs. < 35% [22]	OR, 1.10 (0.43 to 2.81)	0.80	Changing the TTR cut point did not alter the association with rejection
	TTR ≥ 60% vs. < 60% [23]	OR, 0.81 (0.37 to 1.79)	0.60	
**Restricted analysis (months 6–12 only): IPV measures on rejection**^b^	SD (per unit)	OR, 1.26 (0.94 to 1.83)	0.20	IPV metrics remained not significantly associated with rejection
	CV% (per 1% increase)	OR, 1.01 (0.99 to 1.04)	0.30	
	TTR (per 1 percentage point)	OR, 1.00 (0.99 to 1.02)	0.60	
**Induction regimen effect on IPV/exposure metrics**^c^ Basiliximab + methylprednisolone vs. ATG + methylprednisolone	CV%	*β*, 0.39 (− 6.9 to 7.7)	> 0.90	No effect of induction type on IPV or exposure metrics
	TTR	*β*, − 1.2 (− 13.0 to 10.0)	0.80	
	Tacrolimus concentration, ng/mL	*β*, − 0.18 (− 0.83 to 0.47)	0.60	
	Dose-normalized concentration, ng/mL/mg	*β*, 0.13 (− 0.53 to 0.26)	0.50	

^a^Logistic regression analysis

^b^Linear regression analysis

^c^Linear regression for IPV metrics (CV%, SD) and TTR and mixed linear regression for exposure metrics (tacrolimus concentrations and dose-normalized concentrations)

*ATG*, antithymocyte globulin; *CI*, confidence interval; *CV*, coefficient of variation; *DNC*, dose-normalized concentration; *IPV*, intrapatient variability; *OR*, odds ratio; *SD*, standard deviation; *TTR*, time in therapeutic range

## Discussion

This study aimed to investigate the association between intrapatient variability (IPV) of tacrolimus exposure and the risk of acute rejection in pediatric transplant patients. In particular, we analyzed the effects of fluctuating troughs, standard deviations (SD), dose-normalized concentrations (DNC), and coefficient of variation (CV%) of troughs, as well as the percentage of time in therapeutic range (TTR), in 100 pediatric transplant patients. Among the studied patients, we found a strikingly high rejection rate of 46%, with a mean of 1.3 ± 2.0 rejection events per patient during 1 year of follow-up, which represents a relatively high incidence compared with previously published pediatric data [24, 25]. Several factors may account for this finding. Although patients with documented nonadherence were excluded, we were unable to fully capture all variations in adherence between clinic visits. The risk of rejection may also be increased by perioperative recurrent blood transfusion, primarily due to HLA sensitization despite ongoing immunosuppression. Although the use of leukoreduced, irradiated, or washed blood products reduces this risk, it does not eliminate it, as reported in previous studies [26–29]. Additionally, resource limitations in our setting necessitate less frequent monitoring after the first post-transplant month, which may impact the early detection of variability. Most of the transplant recipients in our tertiary center had 3 to 4 HLA mismatches (50.8%), which may increase the immunological risk. Furthermore, pharmacogenetic variations in tacrolimus metabolism, though not assessed in this study, may have contributed to the observed variability [30, 31].

Only CV% demonstrated significant differences in patients who experienced rejection (40.2 ± 17.0% vs. 34.4 ± 16.1%, *p* = 0.031). Alternatively, TTR, SD, and DNC failed to show a significant association with biopsy-proven rejections in the current study. This suggests that the magnitude of variability has a more pronounced effect compared to the duration of variability or dose modifications post-transplantation. It is essential to interpret TTR estimates with caution, as this metric assigns equal weights to both over- and under-exposures of tacrolimus outside the target ranges across different time intervals. Interestingly, our analysis showed that patients who experienced acute rejection spent significantly more time below the therapeutic range (*p* = 0.025), suggesting that under-exposure, rather than overall variability, may be the primary risk for rejection. This finding may explain why TTR alone did not predict rejection: patients with supratherapeutic levels (potentially protective against rejection) were analyzed together with those experiencing subtherapeutic levels (at risk for rejection).

Compared to SD, our findings showed that CV% presented with a smaller variance, which explains the statistically significant differences among rejection groups. In CV% estimation, the smaller variance estimate is related to normalizing the shared random effects between both SD and mean trough concentrations measured within an individual over the follow-up time. After adjustment for confounders and time-dependent effects in the multivariate mixed logistic model for rejection, our findings suggested that higher dose-normalized concentrations are associated with significantly higher rejection rates only at 1–3 months post-transplantation.

In line with the current findings, several studies showed significant associations between increasing CV% and the risk of rejection. In a study investigating the association between different measures of IPV of both tacrolimus and MMF in 46 pediatric transplant patients, the authors demonstrated that a higher tacrolimus CV% was significantly associated with an increased risk of acute rejection. Interestingly, this relationship was not significant with MMF, suggesting that the CV% is a unique rejection indicator for tacrolimus [32]. Several later studies confirmed this relationship between CV% and rejection risk in pediatric transplant patients [8, 33].

Surprisingly, our findings suggested no significant association between TTR and the incidence of graft rejections, in contrast to the existing literature [16]. Similar findings were suggested by Kao et al. who conducted a retrospective study involving 106 lung transplant recipients. Their findings suggested no significant association between tacrolimus TTR and acute rejection of any grade [34]. On the contrary, several studies have reported a significant protective effect of higher TTR on rejection risks in both pediatric [35] and adult kidney transplant recipients [22]. It is important to highlight that the relationship between TTR and rejection risk is inconsistent and time-dependent. In a retrospective study involving 90 lung transplant recipients, there was no significant difference in tacrolimus TTR between the rejection and non-rejection groups. However, in patients who developed rejection after 1 month, tacrolimus TTR was significantly lower than in the non-rejection group [36]. Other potential reasons for the conflicting evidence of the association between TTR and rejection risk may be attributed to differences in populations, definitions of rejection, and TTR cutoffs used within the individual studies.

Comparing IPV metrics over different time intervals post-transplantation showed statistically non-significant differences despite a consistent trend of decreasing CV% and SD measures over time. This decrease reflects both concentration and dose stabilization of tacrolimus therapy; however, statistical significance is probably masked by the significant inter-individual variability of these IPV measures.

Another important finding from the current study is the time-dependent association between IPV and tacrolimus levels and the rejection risk. In the final multivariate mixed logistic model for rejection risk, we found that higher DNC were significantly associated with an increased risk of rejection at 1–3 months post-transplantation (OR 2.69, *p* = 0.010), but not after 3 months. We thought that this relationship could be accounted for based on the changed adherence pattern and exposure precision over time. During longer-term outpatient follow-up, adherence to twice-daily tacrolimus may decline in some patients, potentially increasing variability in trough concentrations. In our cohort, we excluded patients with objective evidence of nonadherence to focus on pharmacokinetic variability, as measured by IPV/exposure metrics, which accurately reflects the true variability and its association with the risk of acute rejection. However, we cannot exclude the possibility of adherence variations that may contribute to increasing IPV over time, particularly after the intensive monitoring period of the first 3 months. Since our study analyzed routinely measured trough levels, which are more frequent during the acute post-transplantation phase compared to later time intervals, the precision of IPV metrics in long-term settings could not be elicited through the current work.

In current clinical practice, we schedule therapeutic drug monitoring (TDM) of tacrolimus to identify whether a patient falls within the therapeutic range of tacrolimus or not. This helps to inform subsequent dosing modifications in both acute and long-term therapy. However, based on the current findings, we strongly recommend further utilizing this TDM data to calculate the individual IPV in tacrolimus trough levels, with a strong preference for both CV% and DNCs. Patients with larger IPV metrics are recommended to follow strict monitoring plans, with more frequent TDM measurements and clinic visits to mitigate their increased risk of rejection. Ultimately, these recommendations pave the way for both short- and long-term individualized management plans, which we thought would be associated with improved clinical outcomes.

To the best of our knowledge, this is the first study to investigate the association between different metrics for IPV in tacrolimus exposure and acute rejection in pediatric kidney transplantation patients. The study was carried out at one of Egypt’s largest pediatric kidney transplant centers, which suggests the generalizability of the current findings.

However, several limitations need to be acknowledged. The dependence on routinely measured tacrolimus through concentrations extracted from electronic health records subjected our findings to information bias, which limits the validity of the findings. Due to the sparse sampling per subject, we utilized a linear interpolation method for estimating the individual profiles of tacrolimus trough levels, which contributes to the current uncertainty of the concluded relationships. However, we mitigated these limitations by restricting the data collection to individuals with complete tacrolimus profiles. The utilized Rosendaal interpolation method was widely adopted for estimating TTR in several previous studies [37, 38]. Also, the findings of the current study should be interpreted with caution due to substantial sources of variability that could potentially mask the true relationship between tacrolimus IPV parameters and rejection risk. In particular, high variability in the estimated CV% and SD across different time intervals, combined with changing target levels throughout the post-transplantation period, could influence potential associations with 1-year rejection risk. Although we employed several methodological approaches to address these limitations, including mixed-effects modeling to account for inter-individual variability, time-stratified analyses adjusted for sampling frequency, and the Rosendaal method to estimate daily exposure, which accounted for the reduced sampling after 1 month, our findings require validation in larger multicenter studies.

## Conclusions

Close attention to tacrolimus exposure is imperative in minimizing the risk of acute rejection after pediatric kidney transplantation. Time below the therapeutic range is more important than time in the therapeutic range for assessing the rejection risk. The CV% of tacrolimus concentration represents a more useful tool than TTR to be considered alongside individual tacrolimus trough levels to minimize the risk of rejection. The DNC of tacrolimus can be used, but not alone as a useful marker for detecting acute rejection, especially from months 1 to 3 in post-transplant periods. Special attention is warranted for therapeutic drug monitoring, especially after the first 3 months post-transplant, due to the increased risk of rejection compared to earlier stages. Future larger prospective studies are warranted to validate the findings of the current work. Furthermore, the effects of genotypes in tacrolimus metabolizing enzymes and concomitant medications need to be investigated.

## Supplementary information

Below is the link to the electronic supplementary material.

Graphical abstract (PPTX 118 kb)

## Data Availability

The datasets used and/or analyzed during the current study are available from the corresponding author on reasonable request.

## Abbreviations

ABMR: Antibody-mediated rejection
ATG: Antithymocyte globulin
CNI: Calcineurin inhibitor
CV: Coefficient of variation
CMV: Cytomegalovirus
C/D ratio: Concentration/dose ratio
DNC: Dose-normalized concentrations
ESPNT: Egyptian Society of Pediatric Nephrology and Transplantation
EMIT: Enzyme multiplied immunoassay technique
FSGS: Focal segmental glomerulosclerosis
HLA: Human leukocyte antigen
IQRs: Interquartile ranges
IPV: Intrapatient variability
MPR: Medication possession ratio
MMF: Mycophenolate mofetil
SD: Standard deviation
TCMR: T-cell-mediated rejection
TTR: Time in therapeutic range
TDM: Therapeutic drug monitoring
