# Supplementary material for: Influence of intrapatient variability in tacrolimus trough levels on acute rejection in pediatric kidney transplant recipients
Source: Pediatr Nephrol. 2025 Dec 22;41(5):1477–88. doi: 10.1007/s00467-025-07028-1 (PMC13009090; doi:10.1007/s00467-025-07028-1)
Supplement: Supplementary file 1 — Graphical abstract (PPTX 118 kb) [file 467_2025_7028_MOESM1_ESM.pptx]

## Slide 1
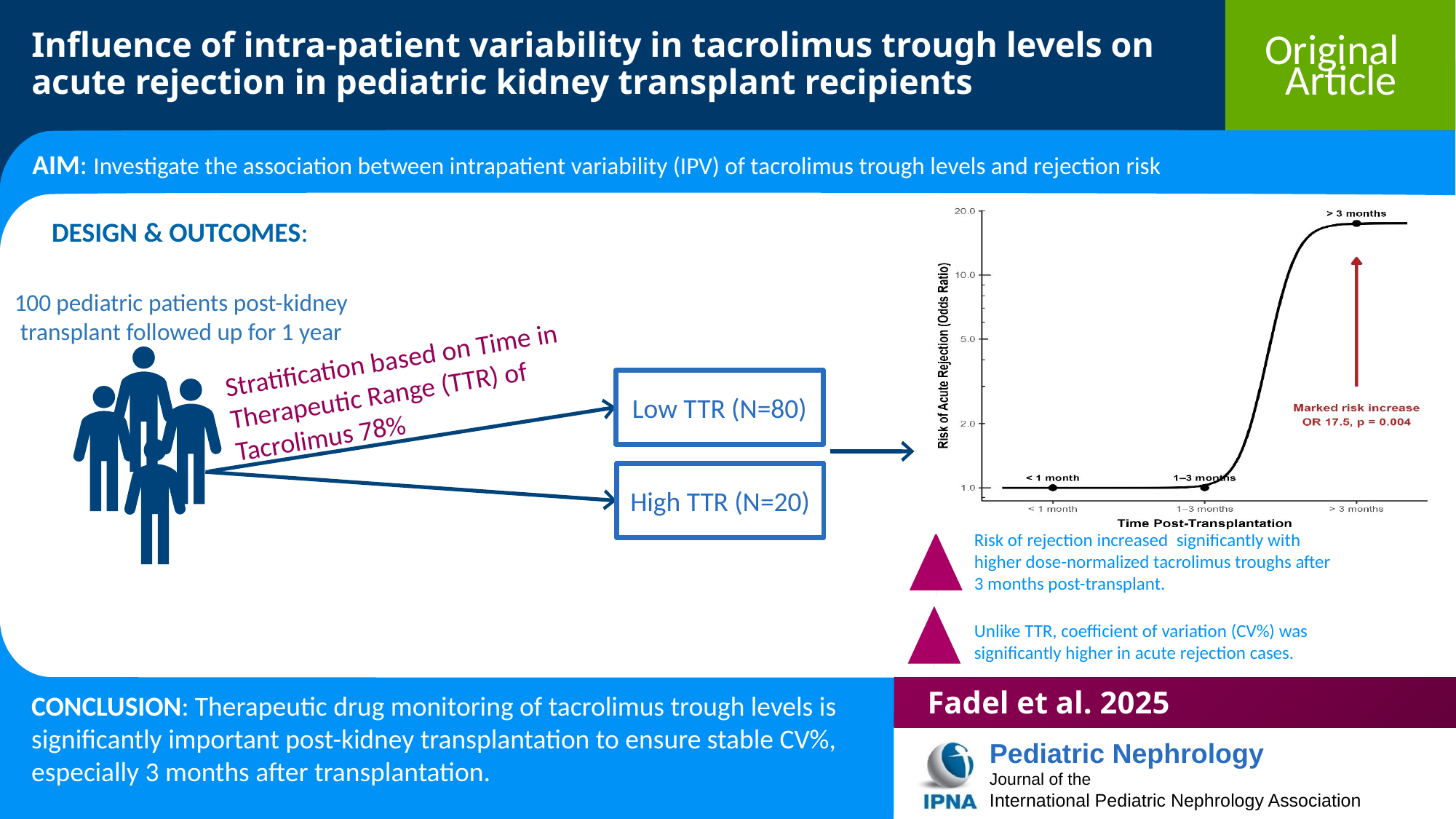

Influence of intra-patient variability in tacrolimus trough levels on
acute rejection in pediatric kidney transplant recipients
AIM: Investigate the association between intrapatient variability (IPV) of tacrolimus trough levels and rejection risk
DESIGN & OUTCOMES:
100 pediatric patients post-kidney transplant followed up for 1 year
Stratification based on Time in Therapeutic Range (TTR) of Tacrolimus 78%
Low TTR (N=80)
High TTR (N=20)
Risk of rejection increased significantly with higher dose-normalized tacrolimus troughs after 3 months post-transplant.
Unlike TTR, coefficient of variation (CV%) was significantly higher in acute rejection cases.
Fadel et al. 2025
CONCLUSION: Therapeutic drug monitoring of tacrolimus trough levels is significantly important post-kidney transplantation to ensure stable CV%, especially 3 months after transplantation.
